# Identification of Group II Intron RmInt1 Binding Sites in a Bacterial Genome

**DOI:** 10.3389/fmolb.2022.834020

**Published:** 2022-02-25

**Authors:** María Dolores Molina-Sánchez, Fernando Manuel García-Rodríguez, Eduardo Andrés-León, Nicolás Toro

**Affiliations:** ^1^ Structure, Dynamics and Function of Rhizobacterial Genomes, Estación Experimental del Zaidín, Department of Soil Microbiology and Symbiotic Systems, Spanish National Research Council (CSIC), Granada, Spain; ^2^ Bioinformatics Unit, Institute of Parasitology and Biomedicine “López-Neyra” (IPBLN), Spanish National Research Council (CSIC), Granada, Spain

**Keywords:** group II intron, RmInt1, IEP, ribonucleoprotein (RNP), ChIP-seq analysis

## Abstract

RmInt1 is a group II intron encoding a reverse transcriptase protein (IEP) lacking the C-terminal endonuclease domain. RmInt1 is an efficient mobile retroelement that predominantly reverse splices into the transient single-stranded DNA at the template for lagging strand DNA synthesis during host replication, a process facilitated by the interaction of the RmInt1 IEP with DnaN at the replication fork. It has been suggested that group II intron ribonucleoprotein particles bind DNA nonspecifically, and then scan for their correct target site. In this study, we investigated RmInt1 binding sites throughout the *Sinorhizobium meliloti* genome, by chromatin-immunoprecipitation coupled with next-generation sequencing. We found that RmInt1 binding sites cluster around the bidirectional replication origin of each of the three replicons comprising the *S. meliloti* genome. Our results provide new evidence linking group II intron mobility to host DNA replication.

## Introduction

Group II introns are considered to be ancient genetic elements present in the genomes of Eubacteria, Archaebacteria, and the organelles of some eukaryotes (Ferat and Michel, 1993; Toro, 2003; Lambowitz and Zimmerly, 2004). They have attracted considerable interest due to their role in driving eukaryotic evolution as the putative ancestor of spliceosomal introns, telomerase, and non-LTR retroelements (Martin and Koonin, 2006; Lambowitz and Belfort, 2015; Zimmerly and Semper, 2015; Novikova and Belfort, 2017; Haack and Toor, 2020), but their properties have also been exploited in the development of powerful biotechnological tools (Mohr et al., 2013; Enyeart et al., 2014; Zhao et al., 2018; Belfort and Lambowitz, 2019).

Group II introns are self-splicing RNAs and mobile retroelements generally consisting of a structurally conserved RNA and a multidomain reverse transcriptase protein (the intron-encoded protein, IEP), which interact with each other to form a ribonucleoprotein (RNP) particle facilitating intron excision and mobility reactions (Lambowitz et al., 1999; Saldanha et al., 1999; Lambowitz and Zimmerly, 2011). The ribozyme consists of six double-helical RNA domains docked around the complex domain I to form a Y-shaped structure (Agrawal et al., 2016; Qu et al., 2016). The group II intron IEP is encoded by domain IV and typically consists of four functional domains: A reverse transcriptase (RT), a maturase (X), a DNA-binding domain (D) and an endonuclease (En) domain (San Filippo and Lambowitz, 2002). Under physiological conditions, both the RT and X domains form contacts with several intron RNA domains (DIV, DII, DIII and DVI) to promote intron folding and splicing (Wank et al., 1999; Zhao and Pyle, 2017). Intron excision occurs by means of two transesterification reactions resulting in ligated exons and several forms of excised introns (lariat, circular, and linear, depending on biological determinants and host environment, Monat and Cousineau, 2020). After splicing, the IEP remains bound to the excised lariat RNA, forming the RNP complex, which performs the mobility reaction via an RNA intermediate (Cousineau et al., 1998; Martínez-Abarca et al., 2004).

**FIGURE 1:**
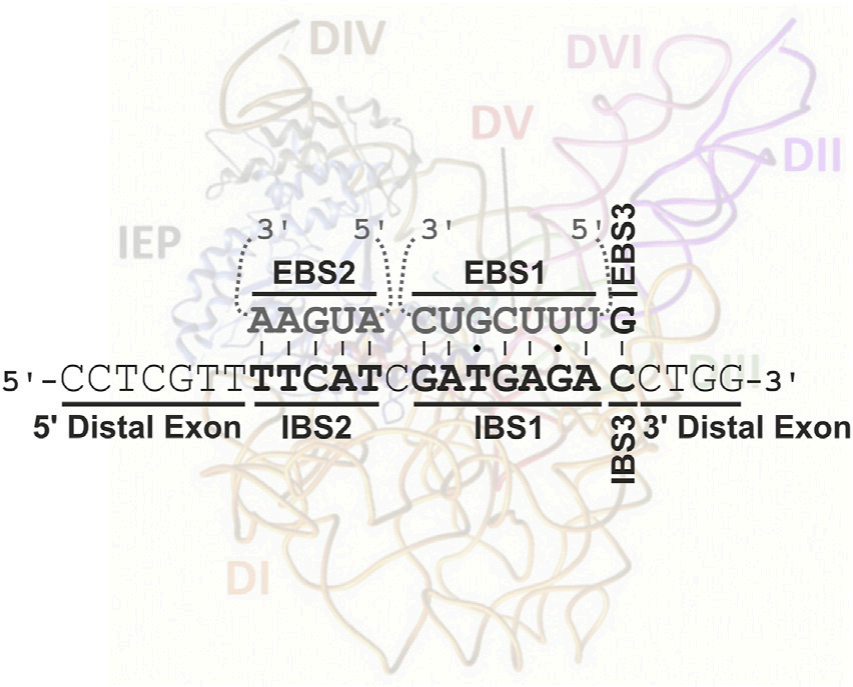
Interaction between RmInt1 and its natural DNA target in ISRm2011-2. The intron binding sites (IBSs) in the DNA target (in bold) base-pair with the exon binding sites (EBSs) in the intron RNA (in gray). These base-pairings extend from positions −13 to +1 of the intron insertion site. An intron RNP complex, adapted from Novikova and Belfort, 2017, is also shown, to illustrate the distal interaction of the IEP at positions G-16, T-15 and G+4.

Group II introns are mobile through a mechanism known as target-primed reverse transcription (TRPT) (Yang et al., 1998; Lambowitz and Zimmerly, 2011). Intron RNPs are widely thought to bind DNA nonspecifically before scanning the DNA for an intron-less target locus (retrohoming) or low-frequency ectopic sequences (retrotransposition) (Aizawa et al., 2003). Homing-site recognition involves an interaction of the C-terminal DNA-binding domain of the IEP with a small number of specific bases in the distal 5′and 3′exon regions of the DNA target site (Guo et al., 1997; Singh and Lambowitz, 2001), but principally via three base-pairing interactions between the intron and exon binding sites (EBS1/IBS1, EBS2/IBS2, and EBS3/IBS3 or δ-δ′) largely responsible for DNA target specificity (Figure 1; Mohr et al., 2000; Jiménez-Zurdo et al., 2003). The intron RNA can then insert itself into one strand of a DNA target site by reverse splicing, and the En domain of the IEP cleaves the opposite strand. Retrohoming proceeds by reverse transcription of the inserted intron RNA to generate a cDNA, using the 3′-end generated by IEP cleavage, with the cellular machinery then resolving incorporation into the host genome (Coros et al., 2008; Coros et al., 2009; Nisa-Martínez et al., 2016). However, almost half the bacterial IEPs lack En domains (Novikova et al., 2014), and the corresponding RNPs must therefore invade their single-stranded DNA targets and then make use of alternative priming strategies for the reverse transcription reaction (Ichiyanagi et al., 2002; Zhong and Lambowitz, 2003; Martínez-Abarca et al., 2004).

One of the best characterized type IIB introns is RmInt1, an intron of the IIB3/D class found in *Sinorhizobium meliloti* (Martínez-Abarca et al., 1998). Despite the absence of an En domain, RmInt1 is an efficient mobile element that integrates into the ISRm2011-2 insertion sequence, which is highly abundant and present in diverse *Rhizobium* species (Martínez-Abarca et al., 2000; Fernández-López et al., 2005). Two retrohoming pathways have been described for RmInt1 mobility: A preferred pathway in which the intron RNA reverse splices intro single-stranded DNA at the replication fork using the nascent lagging DNA strand to prime reverse transcription, and another, less efficient pathway involving retrohoming into the leading strand template (Martínez-Abarca et al., 2004). Moreover, the colonization of the *S. meliloti* genome by the RmInt1 intron is also biased towards RNA insertion into the lagging DNA strand template, even though target sites located on the leading strand template are sometimes invaded, albeit less frequently (Nisa-Martínez et al., 2007). A recent work demonstrated that RmInt1 homing site selection and reverse splicing into the target locus seem to be facilitated during DNA replication by interaction of the IEP with a replicative protein, DnaN, the *β*-sliding clamp (García-Rodríguez et al., 2019). Finally, this intron has been reprogrammed to disrupt both plasmid-borne and chromosomal genes with a high level of efficiency (García-Rodríguez et al., 2011; García-Rodríguez et al., 2014).

Looking to the potential use of RmInt1 as a biotechnological tool, it is crucial to describe off-target insertions that could have highly deleterious effects on the host cell. Here, we investigated the potential binding of RmInt1 RNPs throughout *S. meliloti* genomic DNA *in vivo* by chromatin-immunoprecipitation coupled to next-generation sequencing (ChIP-Seq). We observed a preferential binding around the origin of replication, slightly biased through the template for the lagging strand synthesis during replication. This preference could be related to chromatin accessibility, but we cannot rule out it could be due to the presence of DnaN at the replication fork.

## Methods

### Strains and Growth Conditions

*S. meliloti* RMO17 (Villadas et al., 1995) harboring the different plasmids was cultured in TY medium supplemented with kanamycin (200 μg ml^−1^) at 30°C. Rhizobial bacteria were transformed by triparental mating, with *E. coli* containing the different plasmids. Chromatin immunoprecipitation requires the addition of formaldehyde, which is highly toxic to cells. We therefore assessed the permeability of the cells to formaldehyde and their response to the addition of this chemical before initiating the experiment (Davis et al., 2011). Cultures were grown to an OD_600_ of 0.4, when they were split between two flasks, with formaldehyde (1%) added to one, and a mock treatment (37 mM phosphate buffer) added to the other. Growth was monitored by measuring the OD_600_ at various time points. Growth had stopped after 25 min for the cells incubated with formaldehyde, whereas the bacteria in the mock treatment flask continued to grow exponentially (Supplementary Figure S1). We therefore limited the duration of treatments with this crosslinker to 20 min.

### Construction of Epitope-Tagged IEP Plasmids

**FIGURE 2:**
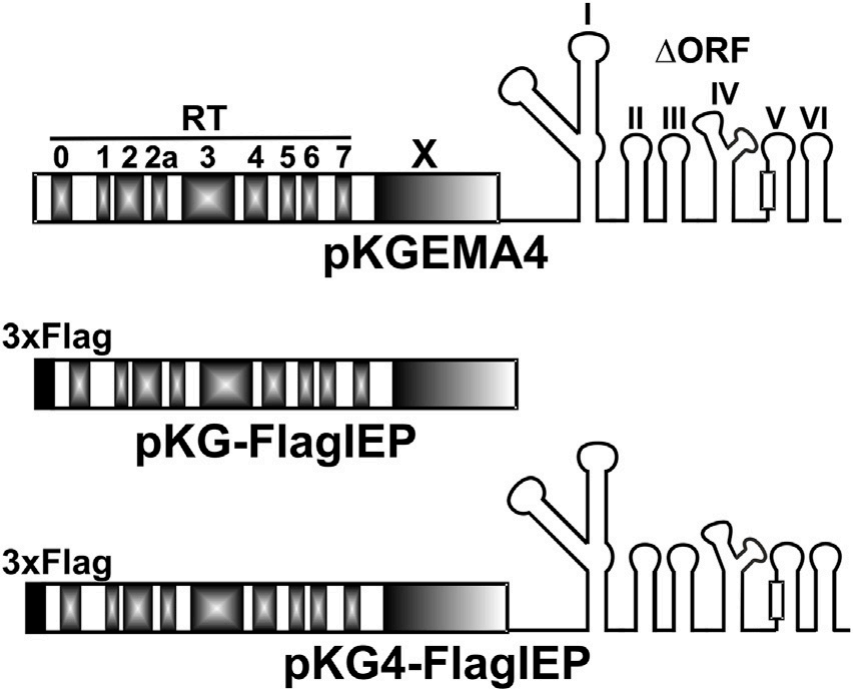
Intron constructs used in the ChIP-Seq experiments. pKGEMA4 contains the wild-type IEP sequence followed by the intron ribozyme with a partial deletion in domain IV, and gives rise to biochemically active RNPs (Nisa-Martínez et al., 2007). Different domains in the IEP are shown in gray. The square in domain V represents the catalytic triad. pKG-FlagIEP encodes the N-tagged IEP with the 3xFLAG epitope (black square) but no intron RNA. Functional RNPs are also produced from pKG4-FlagIEP, but with a 3xFLAG epitope label at the N-terminal end of the IEP.

In the ChIP experiments, three plasmid constructs were used that express the RmInt1 Flag-IEP or Flag-tagged RNPs under the constitutive kanamycin resistance gene promoter: A plasmid (pKG4_FlagIEP) expressing RmInt1 RNPs tagged with a 3xFLAG epitope at the N-terminus of IEP, a similar plasmid construct (pKG_FlagIEP) expressing the 3xFLAG N-tagged IEP but lacking the ribozyme component of the intron, and the pKGEMA4 plasmid (Nisa-Martínez et al., 2007) expressing untagged active RNPs as a negative control (Figure 2). Previous works using pKGEMA4-derivative plasmids have never shown any negative effect in cell viability (Nisa-Martínez et al., 2007; García-Rodríguez et al., 2014). pKG4_FlagIEP was obtained by inserting a fragment consisting of two annealed oligonucleotides with cohesive ends containing a 3xFLAG epitope sequence into an engineered pKGEMA4 plasmid (pKGEMA4NB, containing an *Nde*I site at the ATG of the IEP): 5′3xflag (5′-CTA​GTG​GAA​ACA​GGA​TGG​ACT​ACA​AAG​ACC​ATG​ACG​GTG​ATT​ATA​AAG​ATC​ATG​ACA​TCG​ATT​ACA​AGG​ATG​ACG​ATG​ACA​AGC​A-3′) and 3′3xflag (5′-TAT​GCT​TGT​CAT​CGT​CAT​CCT​TGT​AAT​CGA​TGT​CAT​GAT​CTT​TAT​AAT​CAC​CGT​CAT​GGT​CTT​TGT​AGT​CCA​TCC​TGT​TTC​CA-3′). pKG_FlagIEP was obtained by deleting the ΔORF from pKG4_FlagIEP by cleavage at the *Sac*I restriction sites flanking the ribozyme. Biochemical activities were assayed as previously described (Supplementary Figure S2) (García-Rodríguez et al., 2019).

### Chromatin Immunoprecipitation

ChIP experiments were performed as previously described (Spencer et al., 2003; O’Geen et al., 2010; Bonocora and Wade, 2015; Pini et al., 2015). Briefly, cells in early exponential growth phase (50 ml, OD_600_ of 0.4) were crosslinked by incubation in 1% formaldehyde in 10 mM sodium phosphate (pH 7.6) for 20 min at 30°C on an orbital shaker. Triplicate cultures were set up. Crosslinking was stopped by rapid cooling of the cells on ice, followed by the addition of cold 125 mM glycine. The cultures were then incubated for 5 minutes at room temperature with shaking. The cells were washed three times in phosphate-buffered saline (PBS) and fast-frozen on liquid nitrogen. Cultures were lysed by incubation with 0.4 mg ml^−1^ lysozyme in IP buffer (50 mM Tris-HCl pH 7.5, 1 mM EDTA, 150 mM NaCl, and EDTA-free protease inhibitor [Roche]). We obtained 0.2-0.5 kb DNA fragments by shearing the DNA by sonication (Branson Digital Sonicator 450) on ice, with 27 bursts of 20 s each (50% duty) at 10% amplitude. The resulting lysates were cleared by centrifugation at 10,000 rpm for 15 min at 4°C. At this point, we reserved 1/20 of the cell lysate as the input sample. Anti-FLAG® M2 magnetic beads (Sigma-Aldrich) were added to the rest of lysate, which was then incubated overnight at 4°C on a rotary shaker. ChIP samples were washed four times in TBS buffer (50 mM Tris-HCl pH 7.5, 150 mM NaCl) for 5 min each, at 4°C. The protein/DNA complexes were eluted in 500 μL freshly prepared ChIP elution buffer (50 mM Tris-HCl pH 7.5, 1 mM EDTA, 150 mM NaCl, supplemented with 150 ng μL^−1^ 3xFLAG peptide) for 1 h at 4°C. The input and ChIP samples were incubated overnight at 65°C to reverse the crosslinking. We monitored the immunoprecipitation efficiency by dot-blot using anti-FLAG antibodies (data not shown). The samples were then successively treated with 100 μg of RNaseA for 2 h at 45°C and 100 μg of proteinase K for 2 h at 55°C. The DNA was purified by phenol extraction followed by ethanol precipitation.

### ChIP Sequencing, Data Alignment and Peak Calling

A total of 18 samples were studied. Nine DNA samples were control (input) samples and the remaining samples corresponded with immunoprecipitated DNA. In turn, each group of nine samples came from bacterial cultures carrying three different plasmid constructs: A plasmid that expresses non-tagged, functional RNPs (pKGEMA4); a vector that produces 3xFLAG-tag IEP (pKG_FlagIEP); or, finally, a construct which generates 3xFLAG-tagged, active RNPs (pKG4_FlagIEP) (Figure 2). Experiments were performed in triplicates.

**FIGURE 3:**
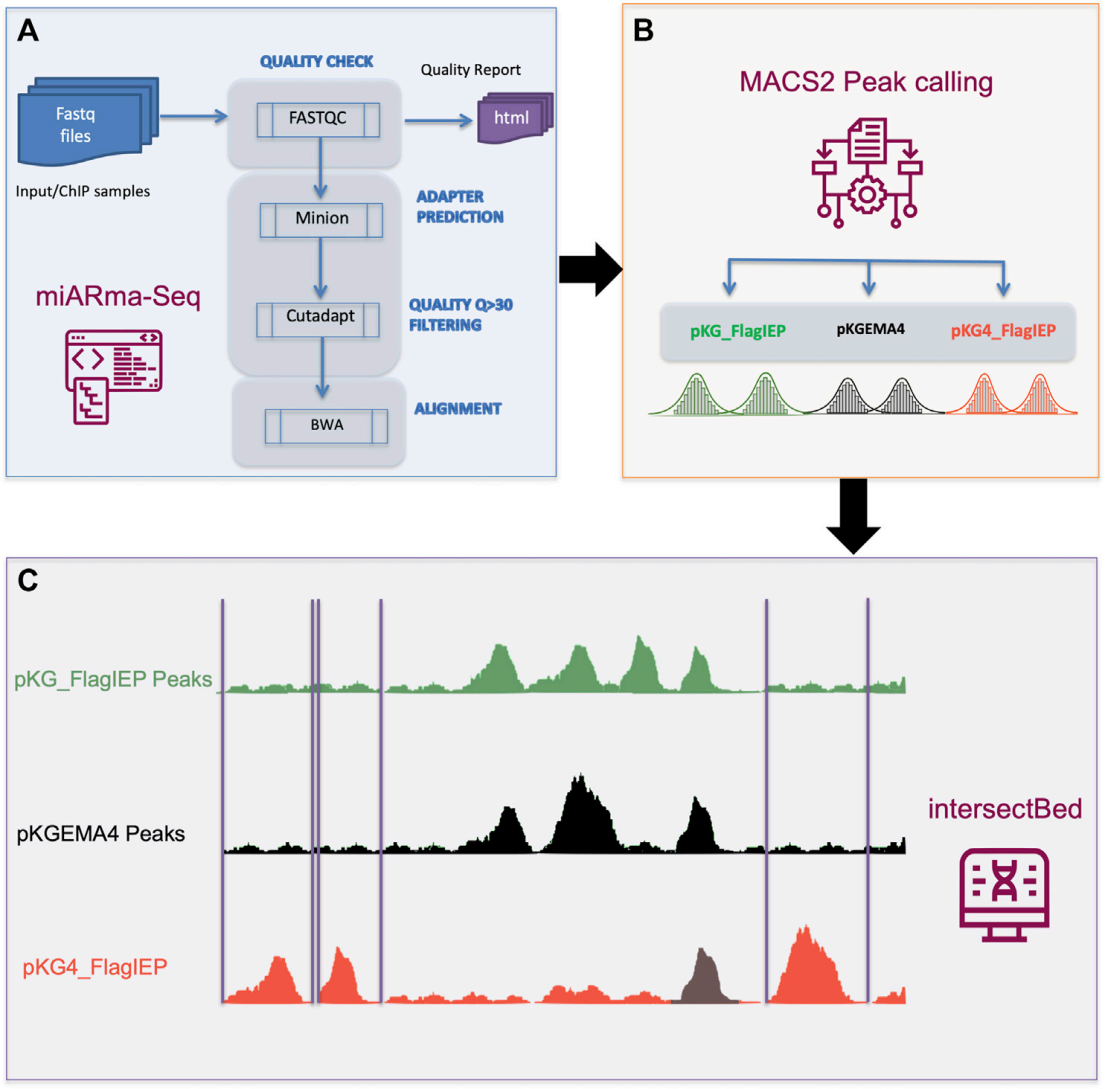
ChIP-Seq data analysis pipeline. **(A)** Quality assessment and samples alignment were performed using the miARma-Seq software. Fastqc 0.11.5 was used to study the overall sequence quality and to identify possible adapter accumulation. Furthermore, we applied minion to identify any possible adapter sequences. As none were found, we used cutadapt to select reads having a Phred score > 30. Next, those sequences were aligned using BWA. **(B)** Resulting files were processed with MACS2 to identify statically reliable peaks (FDR < 0.001) per each group of samples. **(C)** IntersectBed from bedtools were used to identify unique peaks in pKG4_FlagIEP by removing common peaks in pKG_FlagIEP or pKGEMA4.

Subsequently, DNA samples were processed by standard protocols and sequenced at the IPBLN Genomics Unit on an Illumina Nextseq500 with 75-bp single-end reads. Finally, a total of 30 million reads were obtained (on average). Quality assessment and samples alignment were performed using the miARma-Seq software (Andrés-León et al., 2016). In detail, miARma-seq contains all the required software to process most type of NGS samples (Figure 3). In the first step, fastqc V.0.11.5 was applied to gather the overall sequence quality and to identify possible adapter accumulation (Andrews, 2010). The Per base sequence quality and the Per sequence quality scores showed that most of the reads quality were above 30. The Per base sequence content displayed a proportional distribution of the four nucleotides along the 75 bp, besides, no Illumina adapter was found in the adapter content section of the fastqc report. However, we use minion (Davis et al., 2013), a software that predict possible adapter sequences by reading the first nucleotides of the reads searching for a consensus sequence. In our case no consensus sequence was found. Next, we use Cutadapt (Martin, 2011) to filter those reads having a quality score below 30.

Finally, an average of 27 M (>90%) reads per sample were used for the next step. We then aligned all the samples with the *Sinorhizobium meliloti* strain RMO17 reference genome from GenBank (accession numbers CP009144 to CP009146) using default parameters in Burrows-Wheeler Aligner software (Li and Durbin, 2010). On average we obtained an alignment percentage of reads against the reference genome above 97.53%. Aligned files were processed with MACS2 (Zhang et al., 2008) to identify statically reliable peaks. To achieve this, we analyze the three inputs and the three ChIP samples simultaneously in a single MACS2 execution, instead of separated running of the replicates and then combine common peaks. By running the samples together, we can achieve more reliable peaks even with moderately low enrichment (Wilbanks and Facciotti, 2010). Besides, the common parameter in MACS2 used in each of the three group were -f BAM --nomodel --keep-dup all -g 6.7e6. Only peaks statistically significant [LOG_10_ (*q* value) > 3 (FDR < 0.001)] were considered.

The resulting sets of enriched peaks obtained for each of the three plasmid constructs were analyzed with IntersectBed (belonging to the Bedtools suite; Quinlan and Hall, 2010) to obtain the set of common or unique peaks in each set. Once the overlapping peaks between samples from different constructs were removed, we focus to describe the unique peaks in the pKG4_Flag-IEP samples. This was accomplished in two steps: first, intersectBed -a pKG4_FlagIEP -b pKG_FlagIEP -v > file1.bed; and next, intersectBed -a file1.bed -b pKGEMA4 -v > UniquePeaks_ pKG4_FlagIEP.bed). The final bed file contains 321 statistically reliable peaks FDR< 0.001 not shared neither by FlagIEP nor by pKGEMA4.

The raw sequencing data have been deposited in the SRA database with BioProject accession number PRJNA779974.

## Results and Discussion

### Identification by ChIP-Seq of *in vivo* DNA Regions Binding RmInt1 RNPs

Chromatin immunoprecipitation followed by Illumina high-throughput next-generation sequencing (ChIP-Seq) has already been successfully used to identify transcription factors providing a high-resolution snapshot of protein/DNA interactions (Spencer et al., 2003; Park, 2009; Furey, 2012; Bonocora and Wade, 2015). We aim to identify the regions of the *S. meliloti* genome binding RmInt1 RNPs *in vivo* using ChIP-Seq analyses, by introducing various plasmid constructs into RMO17, an intron-less *S. meliloti* strain (Figure 2). pKG4-FlagIEP encoded 3xFLAG-tagged active RNPs (Supplementary Figure S2; Reinoso-Colacio et al., 2015); pKG-FlagIEP expressed the FLAG-tagged IEP but lacking the ribozyme component of the intron; finally, as an IP control, pKGEMA4 encoded untagged functional RNPs (Nisa-Martínez et al., 2007). We constructed 18 libraries corresponding to the input and ChIP samples in triplicate, and we obtained a total of 508,880,560 high-quality reads, which we then mapped back onto the *S. meliloti* RMO17 genome (Supplementary Table S1), reaching around 300-fold genome coverage for each individual library. We found 574 potential binding sites corresponding to the construct producing active RNPs (pKG4_FlagIEP); 276 enriched regions for the construct expressing the IEP alone (pKG_FlagIEP); and, 428 peaks in the untagged control (pKGEMA4) (Supplementary Table S2). Then, we filtered out peaks resulting from IEP interactions in the absence of the ribozyme by comparing the binding sites identified when the 3xFLAG IEP was introduced alone (pKG_FlagIEP) to the peaks observed when the labelled protein was present together with the intron RNA forming active RNPs (pKG4_FlagIEP). Further filtering was performed by removing any peaks found in non-tagged functional RNPs (pKGEMA4), which resulted in 321 distinct DNA fragments appearing only in the FLAG-tagged, functional RNP (pKG4_FlagIEP) output data (Figure 3).

Most ChIP-seq reports consider peaks to be significant for an enrichment ≥ 1.5-2-fold (Myers et al., 2015; Šmídová et al., 2019), and the binding of some transcription factors, generally to specific motifs, results in a 10- to several hundred-fold enrichment (Martínez-Granero et al., 2014). However, the majority of the RmInt1 RNPs binding DNA sequences identified in our study (77%) showed a signal-to-noise ratio [S/N] ranging 1.08-1.14 (Supplementary Figure S3A). A 19% of the identified peaks exhibited a fold-enrichment ratio falling between 1.14 and 1.18, and only a few regions (5) presented enrichment ratios beyond 1.18 up to 1.23. ChIP-seq enrichment after immunoprecipitation is influenced by the strength of the interaction. Furthermore, low-enrichment signals in ChIP-seq experiments may reflect indirect binding to a third counterpart bound to DNA (Furey, 2012). The low ChIP signals in our samples may, therefore, reflect weak interactions of the RmInt1 RNPs with the genomic DNA, or may suggest that DNA binding is dependent on other interacting factors, for instance, DnaN (García-Rodríguez et al., 2019).

*S. meliloti* RMO17 genome contains 13 copies of the natural homing site of RmInt1, ISRm2011-2 (Toro et al., 2014). However, we were unable to detect significant enrichment relative to the input DNA around any of the 13 copies of the insertion sequence by qPCR on ChIP samples (Supplementary Figure S4A). Similar numbers of reads were recruited for all libraries when we considered the full-length sequence of ISRm2011-2 (Supplementary Figure S4B). Nevertheless, some differences at the IS intron insertion site were observed in the immunoprecipitated samples. We scanned the ChIP-seq libraries for three 25 nt sequences: the RmInt1 insertion site (5′-CCT​CGT​TTT​CAT​CGA​TGA​GAC​CTG​G-3′), a sequence located 50 nt upstream (5′-CGA​ACG​GGA​GCG​GCC​CGA​CGT​CGC​C-3′) or a sequence located 50 nt downstream (5′-ACT​GGT​GGG​CTA​CGC​CCC​CTT​CGG​C-3′). We determined the number of sequences contained in each library that aligned with the above described regions, normalizing by the total number of sequences in the corresponding library, as a means of comparing data for different libraries (Supplementary Figure S4B). Interestingly, a significant bias towards a decreased number of recruited sequences containing the insertion site was observed when active RNPs were present, probably due to intron insertion during retrohoming process. Thus, the binding of RmInt1 RNPs to DNA may not be restricted to specific sequence motifs/sequences in agreement with a previously suggested nonspecific attachment of the intron RNPs to DNA (Aizawa et al., 2003).

### Genome-wide Analysis of the Enriched Peak for the Binding of Functional RmInt1 RNPs

*S. meliloti* RMO17 has a tripartite genome comprising a 3.65 Mb chromosome, and two symbiotic megaplasmids: pSymA (1.47 Mb) and pSymB (1.61 Mb) (Toro et al., 2014). We identified 321 regions displaying some binding to RmInt1 active RNPs, ranging from 200–800 bp in length (Supplementary Figure S3B; Supplementary Table S2). Most of the enriched peaks corresponded to chromosomal regions (266), with only a minority of the identified sequences mapping to the megaplasmid pSymA (11) and the pSymB chromid (44). The precise coordination of genome replication, chromosome segregation and cell division is essential for population fitness, and bacteria with multipartite genomes must conserve one copy of each replicon per cell cycle (Frage et al., 2016). Differences in the proportions of regions potentially bound by RmInt1 RNPs cannot, therefore, simply be attributed to differences in copy number or replicon size. They may instead reflect differences in replicon accessibility.

**FIGURE 4:**
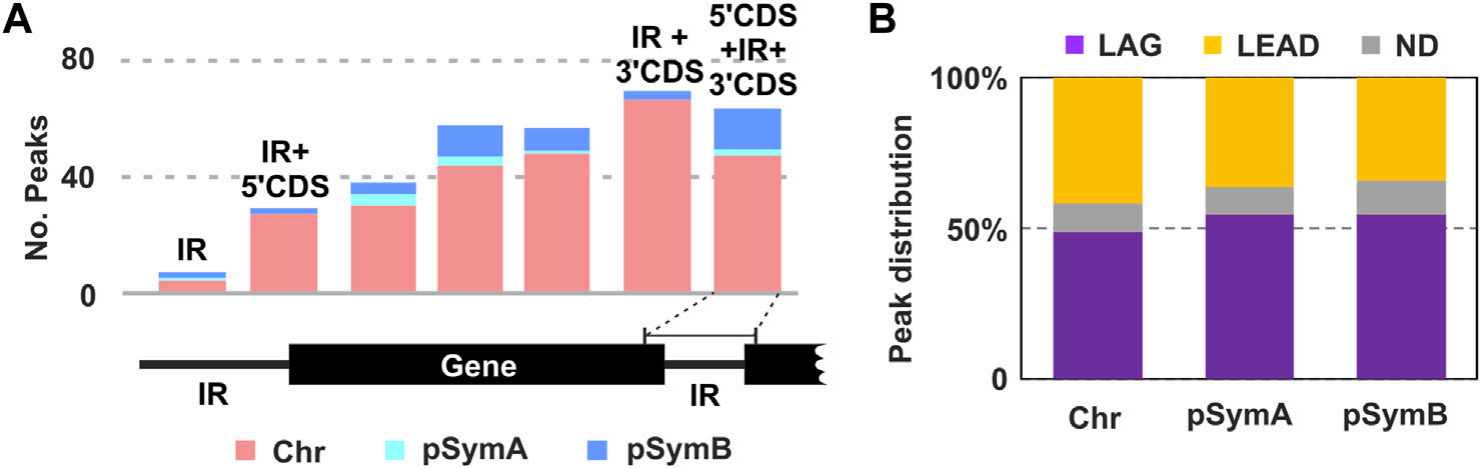
Result of the RmInt1 ChIP-Seq analysis. **(A)** Genomic localization of the identified RmInt1 binding sites. Bar plot showing the number of peaks found in either the coding sequence of a gene (CDS) or the intergenic regions (IR). Counts are itemized by replicon: pink corresponds to binding sites identified in the chromosome; sky blue corresponds to the pSymA megaplasmid; and, the darker blue corresponds to the pSymB chromid. IR + 3′CDS is not included in the 5′CDS+IR+3′CDS. **(B)** The proportions of enriched DNA sequences localizing at the template for the lagging (LAG, purple) and leading (LEAD, yellow) strands at the replication fork are shown as a percentage in a stacked bar graph. Data are presented by replicon. ND, non-determined (gray).

We then considered possible bias in associations with respect to genome architecture. Most of the binding regions identified were located in annotated genes (51), with a certain preference toward the 3′-end of the coding sequence (Figure 4A). We also identified bound DNA fragments in intergenic regions (5), and others extending to the 5′ end of the downstream gene (11), the 3′ end of the upstream gene (27), or to both these positions (37). Again, we observed a higher number of peaks comprising the 3′-end of the gene and the downstream IR compared to the regions containing 5′CDS + IR. Although a certain bias could be considered, more evidences need to be obtained in order to establish a believable correlation between intron biology and RmInt1 RNP binding to DNA at the 3′-end of genes.

Previous studies have reported a preferred insertion site of the RmInt1 ribozyme on the template for lagging strand synthesis during DNA replication (Martínez-Abarca et al., 2004). We wondered whether the binding of RmInt1 RNPs displayed a similar bias genome-wide. According to the annotations of the origin and termination of replication in the genome sequence (Lato and Golding, 2020), we calculated, where possible, the most probable orientation of the binding sequences with respect to the movement of the replication fork (Figure 4B). In general, a certain preference for RmInt1 RNP binding to the template for lagging strand synthesis was detected in all three replicons, consistent with the intron colonization bias observed in nature (Nisa-Martínez et al., 2007). Since DNA transcription and replication are spatiotemporally coordinated in bacteria, head-on collisions between protein machineries during lagging strand synthesis cause replication arrest (Rocha, 2004). Thus, it is tempting to speculate that the increased availability of single-stranded DNA favors binding of En- intron RNPs.

### Mapping DNA-Binding Regions in the *S. meliloti* Genome

**FIGURE 5:**
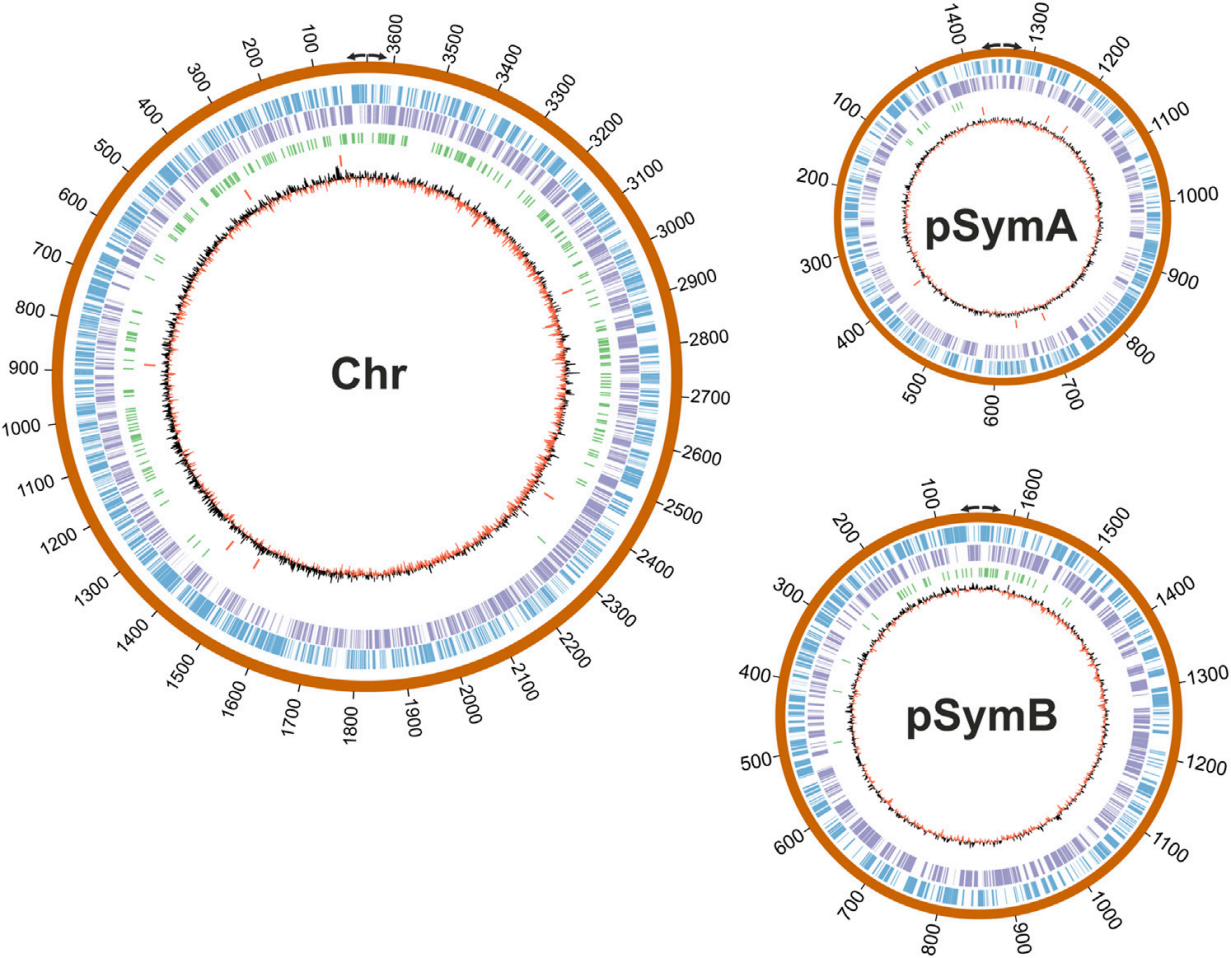
Mapping of the RmInt1 binding sites in the *S. meliloti* RMO17 genome. The three replicons are drawn to scale with CIRCOS. The outer track in blue corresponds to the forward annotated genes (top strand) and the purple circle corresponds to the reverse orientation genes (bottom strand). Identified ChIP-Seq peaks are shown in green and the 13 copies of the natural target sequence of RmInt1 (ISRm20011-2) are indicated using red bars. The skewed GC plot is shown in red/black lines. Divergent arrows indicate the replication origin of each plasmid according to the data published by Lato and Golding (2020).

Next, we wondered whether RmInt1 RNP binding displayed positional preferences in the genome. Then, the 321 enriched regions identified in our study were mapped in *S. meliloti* RMO17 genome. Remarkably, we observed a clear, biased distribution around the replication origins in each of the replicons: The chromosome, pSymA and pSymB (Figure 5). A similar result has been reported for insertion of the mobile *Lactococcus lactis* Ll.LtrB intron at sites clustered near the bidirectional *oriC* in the *E. coli* genome (Zhong et al., 2003). Moreover, in *E. coli*, the location of the wild-type Ll.LtrB retrotranspose around the origin and terminus for chromosomal DNA replication is influenced by growth conditions (Coros et al., 2005). This behavior is not exclusive to group II introns; insertion sequences and transposons also use mechanisms that coordinate transposition and DNA replication (Hu and Derbyshire, 1998; Peters and Craig, 2001).

It had been described that fast-growing bacteria requires simultaneous rounds of replication to achieve their growth rates (Rocha, 2004). Thus, regions close to the replication origin may be overrepresented relative to the regions distal to the replication origin. *S. meliloti* could be considered a moderately fast-growing bacteria with an optimal duplication time around 140 min (De Nisco et al., 2014). If the replication fork moves at about 600-1,000 nt s^−1^ (Rocha, 2004), duplication time should be enough for the completion of the chromosome synthesis in exponential growing *S. meliloti* cell cultures. As mentioned, experiments of segregation timing suggest that DNA replication occurs only once per cell cycle (Frage et al., 2016). Since we observed a bias of the RmInt1 RNPs around the origin of replication, we aim to discard this bias is due to differences in DNA abundance along the replicon. In that sense, we have calculated the coverage for each sample and performed a *t*-test of the mean coverage in control (input) against immunoprecipitated samples. This calculation gave a non-significant *p*-value > 0.05 when considered either the whole replicon or only the first 1,000 nt from the replication origin, indicating that the distribution of DNA reads is similar in both kind of samples (data not shown).

Many host factors, mostly related to the replication machinery, contribute to the retrohoming of group II introns (Smith et al., 2005; Coros et al., 2008; Zhao et al., 2008; Coros et al., 2009; Yao et al., 2013; Nisa-Martínez et al., 2016). One recent study revealed that the RmInt1 IEP and active RNPs interact with DnaN (*β*-sliding clamp), a replicative protein that forms part of the DNA polymerase III complex (García-Rodríguez et al., 2019). An analysis of *S. meliloti* DnaN-mCherry dynamics revealed a strict spatiotemporal localization directly connected with the order of segregation of the three bacterial replicons (Frage et al., 2016). The bidirectional replication of the chromosome occurs first, followed by the megaplasmid pSymA and then by the chromid pSymB (De Nisco et al., 2014; Frage et al., 2016). DnaN-mCherry was found to disperse before the onset of a new cell division event. By contrast to the polar location of the chromosome origin of replication, the replication origins of the megaplamids are subpolar after the completion of segregation. On the other hand, fluorescence microscopy has shown that RmInt1 IEP-EGFP RNPs are mostly dispersed throughout the cell, but that a small proportion localize at several foci within exponentially growing *S. meliloti* RMO17 cells (Nisa-Martínez et al., 2013). Similar results were described for GFP fusions of the Ll.ltrB group II intron (Zhao and Lambowitz, 2005; Beauregard et al., 2006; Zhao et al., 2008). We therefore suggest that interaction with DnaN may control the polar localization of RmInt1 RNPs, and that the subpolar position of the origins of megaplamids influences the binding events around these origins. The preferential location of binding sites around the origin of replication may be related to replicon segregation times.

## Concluding Remarks

Our results provide new evidences that support a connection between intron functionality and host DNA replication. The nonspecific binding of RmInt1 RNPs to *S. meliloti* genomic DNA showed a preference for the template of the lagging strand during replication. Moreover, we observed preferential binding around the origin of replication. These preferences may be related to chromatin accessibility, or the interaction of the RNPs with host replicative proteins, but also binding can be conducted by the intron RNPs distribution into the bacterial cells during DNA replication. These observations need to be considered when reprogrammed introns are used for whole genome knockout approaches.

## Data Availability

The datasets presented in this study can be found in online repositories. The names of the repository/repositories and accession number(s) can be found below: https://www.ncbi.nlm.nih.gov/, PRJNA779974.
