# Supplementary material for: Identification of Group II Intron RmInt1 Binding Sites in a Bacterial Genome
Source: Front Mol Biosci. 2022 Feb 25;9:834020. doi: 10.3389/fmolb.2022.834020 (PMC8914252; doi:10.3389/fmolb.2022.834020)
Supplement: Supplementary file 2 [file DataSheet1.DOCX]

Supplementary Material

# Supplementary Figures and Tables

## Supplementary Figures


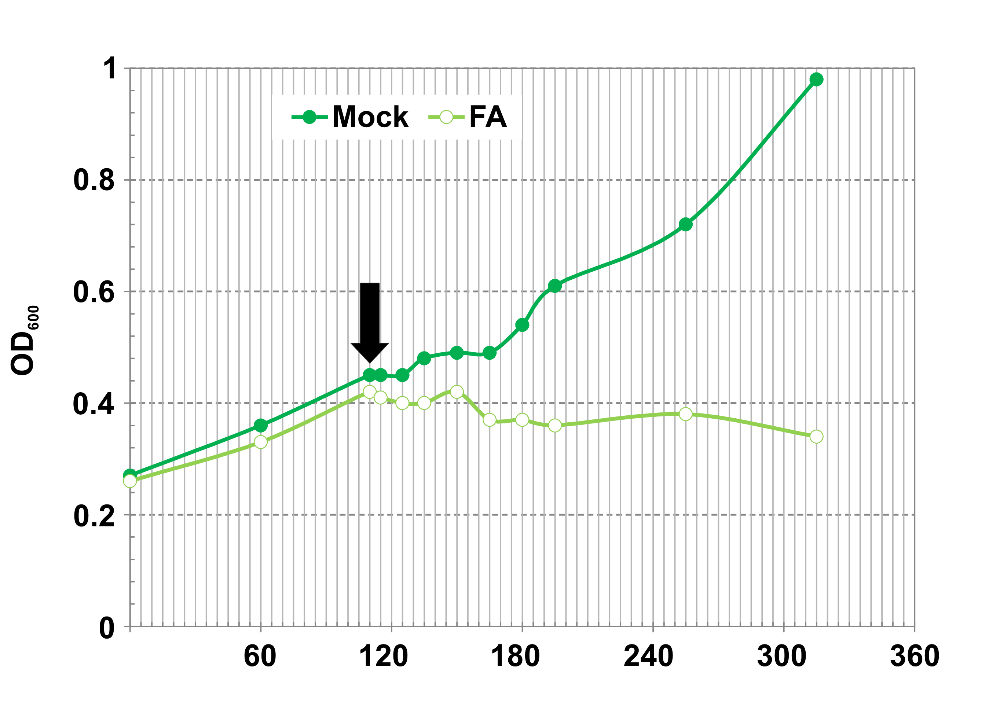


**Supplementary Figure 1.** *S. meliloti* RMO17 growth curve in TY broth supplemented (open circles) or not supplemented (closed circles) with 1% formaldehyde. The black arrow indicates the time at which the treatment was added.


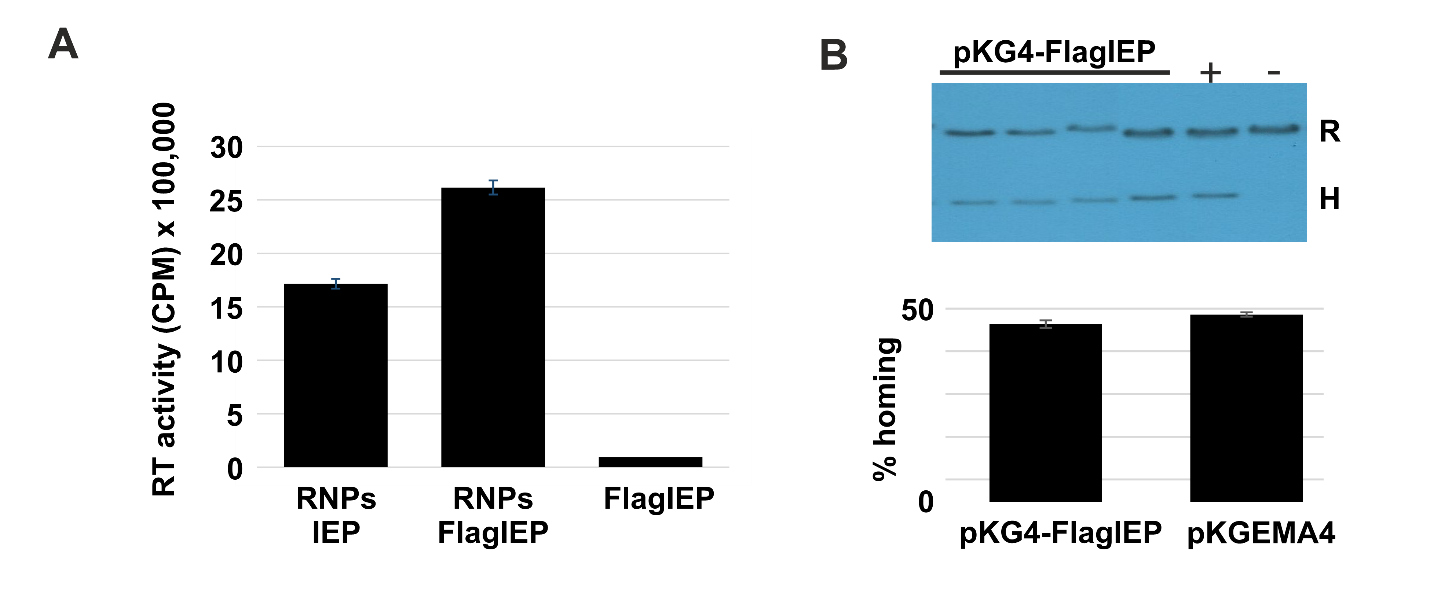


**Supplementary Figure 2.** Biochemical activities of the different constructs used in our study. **(A)** Exogenous reverse transcriptase activity *in vitro* was assayed with poly(rA)/oligo(dT)_18_ and [α-P^32^] dTTP. RT activity is expressed in counts per minute (cpm). The data shown are the means for at least three independent assays and three independent *in vivo* RNP preparations. Error bars represent the standard errors. **(B)** A homing assay involving hybridization with an intron DNA probe is shown in the upper panel. R, recipient plasmid; H, homing product. *S. meliloti* RMO17 containing the recipient plasmid pJB0.6LAG was transformed with the donor construct pKG4_FlagIEP or pKGEMA4 (+). A recipient plasmid lacking the recognition DNA target region (pJBΔ129) was used as a control (-). Graph representing retrohoming efficiency, calculated as the ratio of invaded recipient plasmid to the total recipient plasmid in the cell and expressed as a percentage. The data shown are the means for at least two independent colonies ± standard errors.


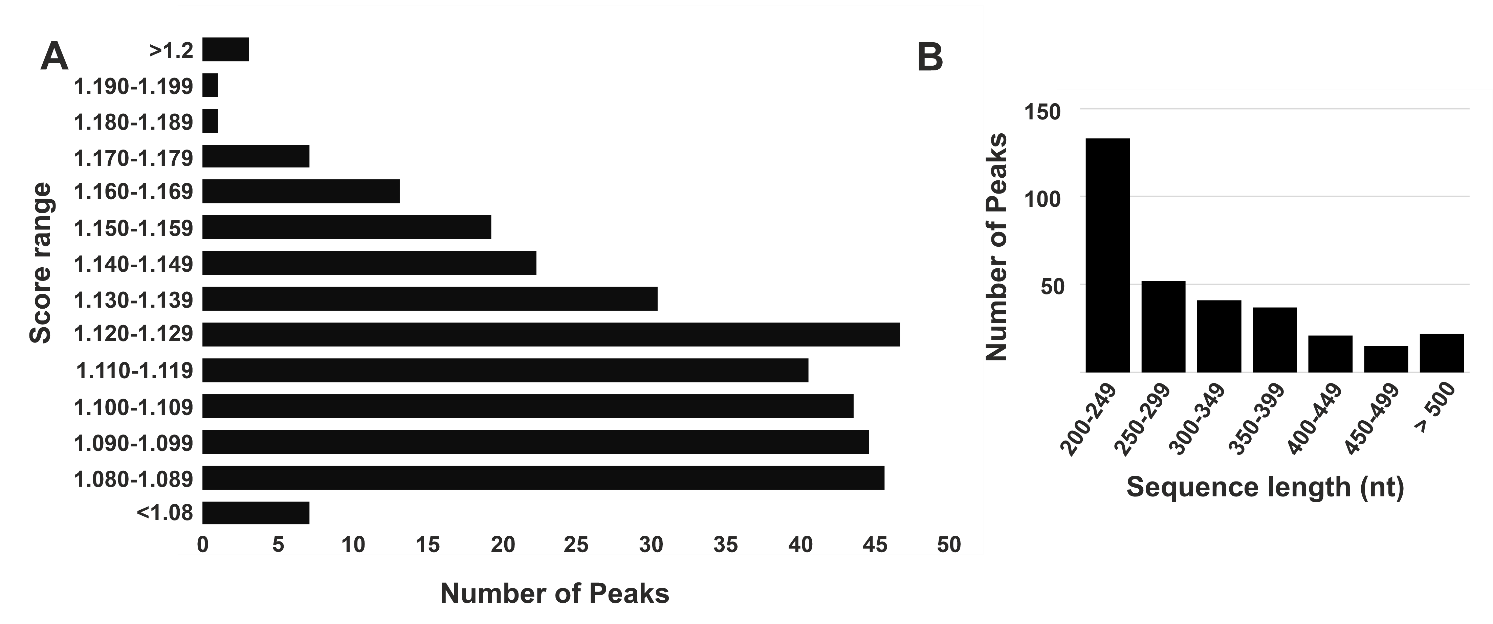


**Supplementary Figure 3.** ChIP-Seq data analysis. **(A)** Bars represent the number of peaks reaching the indicated fold-enrichment score in the ChIP experiment. **(B)** Plot representing the distribution of peaks by sequence length.


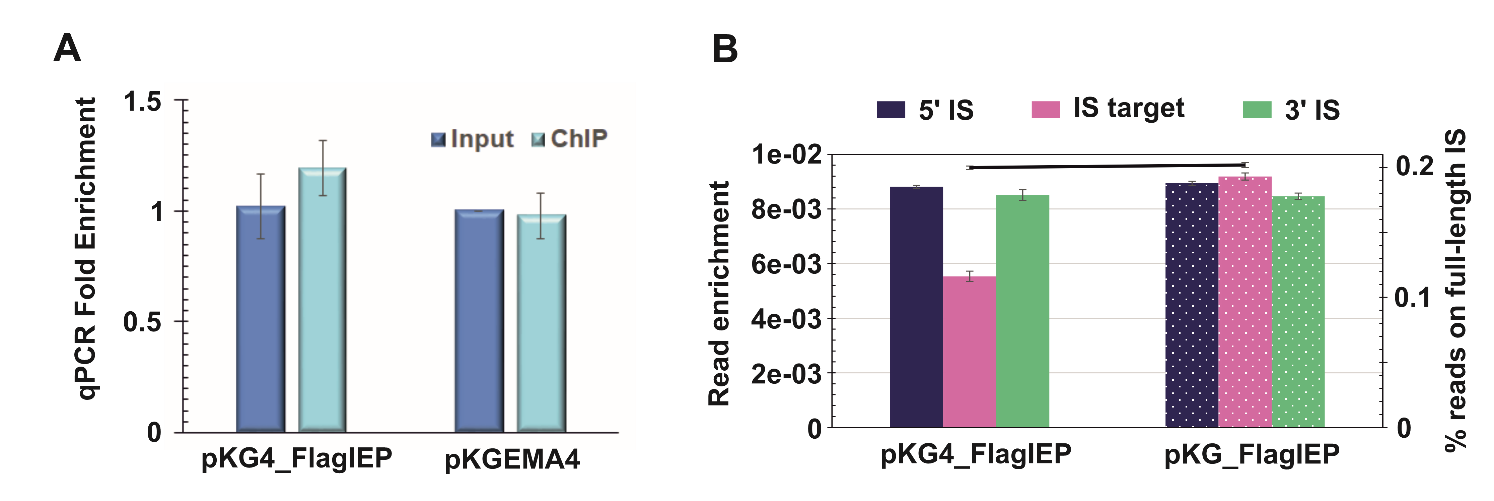


**Supplementary Figure 4.** ISRm2011-2 enrichment in ChIP samples. **(A)** Bars represent the fold-enrichment determined by qPCR. Primers flanking the intron insertion site were used to check IS sequence accumulation in the ChIP samples relative to the input samples. **(B)** The line above the graph corresponds to the percentage of the total number of reads in the ChIP raw data recruited to the full-length ISRm2011-2 sequence with Geneious. Bar graphs represent the normalized number of reads from the ChIP raw data mapping to three 25 nt sequences within the ISRm2011-2: 5’IS located 50 nt from the 5’-end of the RmInt1 insertion target site; IS target is the minimum -20/+5 DNA target identified for RmInt1 insertion; and 3’IS is the sequence localizing to 50 nt from the 3’-end of the IS target site. Reads were normalized according to the total number of reads in each library. Error bars represent the standard errors for the three replicates.

## Supplementary Tables

| Supplementary Table 1. Library information. | | | | | |
| --- | --- | --- | --- | --- | --- |
|  | **ID** | **QUBIT**  **[ng/μl]** | **Size**  **(bp)** | **Molarity**  **(nM)** | **Output M reads** |
| 1 | Input RMO17:pKG4_FlagIEP Col.1 | 7.74 | 607 | 19.3 | 27 |
| 2 | ChIP RMO17:pKG4_FlagIEP Col.1 | 7.05 | 395 | 27 | 31 |
| 3 | Input RMO17:pKG4_FlagIEP Col.2 | 5.58 | 675 | 12.5 | 31 |
| 4 | ChIP RMO17:pKG4_FlagIEP Col.2 | 7.7 | 431 | 27 | 28 |
| 5 | Input RMO17:pKG4_FlagIEP Col.3 | 12.4 | 675 | 27.8 | 22 |
| 6 | ChIP RMO17:pKG4_FlagIEP Col.3 | 10.5 | 439 | 35.2 | 28 |
| 7 | Input RMO17:pKG_FlagIEP Col.1 | 9.11 | 660 | 23 | 25 |
| 8 | ChIP RMO17:pKG_FlagIEP Col.1 | 6.73 | 404 | 25.2 | 26 |
| 9 | Input RMO17:pKG_FlagIEP Col.2 | 1.29 | 600 | 3.25 | 28 |
| 10 | ChIP RMO17:pKG_FlagIEP Col.2 | 10.8 | 400 | 40.9 | 27 |
| 11 | Input RMO17:pKG_FlagIEP Col.3 | 8.21 | 600 | 22.8 | 29 |
| 12 | ChIP RMO17:pKG_FlagIEP Col.3 | 13.2 | 400 | 50 | 27 |
| 13 | Input RMO17:pKGEMA4 Col.1 | 7.3 | 500 | 22.1 | 24 |
| 14 | ChIP RMO17:pKGEMA4 Col.1 | 6.69 | 385 | 26.3 | 26 |
| 15 | Input RMO17:pKGEMA4 Col.2 | 10.1 | 613 | 25 | 29 |
| 16 | ChIP RMO17:pKGEMA4 Col.2 | 11.9 | 400 | 45 | 3 |
| 17 | Input RMO17:pKGEMA4 Col.3 | 22.3 | 613 | 55.1 | 26 |
| 18 | ChIP RMO17:pKGEMA4 Col.3 | 16 | 384 | 63.1 | 34 |
| NEBNext Ultra II (NEB) (Input DNA 10‐50 ng; Adaptor dilution 1:10; no. of PCR cycles= 7)  High output, 75 cycles SE 1X75  Run, total length 40,20 Gb  Run, total reads (PFR) 508,880,560 reads  Quality control: Qubit/BioAnalyzer *High-Sensitivity DNA* | | | | | |
|  | | | | | |

**Supplementary Table 2.** List of the enriched ChIP-Seq peaks in samples with active RmInt1 RNPs. Excel file Supplementary Table 2.
